# An efficient and sensitive chemosensor based on salicylhydrazide for naked-eye and fluorescent detection of Zn^2+^†

**DOI:** 10.1039/c7ra13592k

**Published:** 2018-02-12

**Authors:** Zhanglin Shi, Yayi Tu, Shouzhi Pu

**Affiliations:** Jiangxi Key Laboratory of Organic Chemistry, Jiangxi Science and Technology Normal University Nanchang Jiangxi 330013 PR China pushouzhi@tsinghua.org.cn +86-791-83831996 +86-791-83831996

## Abstract

We reported here the synthesis of a diarylethene with a 2,4-dihydroxybenzoyl hydrazine moiety (1O) for Zn^2+^ recognition. The compound is easy to prepare with a high yield up to 85%. Compound 1O can act as a highly selective and specific fluorescent sensor for Zn^2+^ without interference by other common metal ions. The LOD for Zn^2+^ detection was determined to be 1.28 × 10^−6^ mol L^−1^. Meanwhile, 1O can be used as a naked-eye detector for the Zn^2+^ ion with an obvious color change from colorless to olive. Based on the fluorescent properties of 1O, we constructed a logic circuit with four inputs of the combinational stimuli of UV/vis light and Zn^2+^/EDTA, and one output of fluorescence intensity.

## Abstract 2

A naked-eye and fluorescent detector for Zn^2+^ was synthesized by the combination of salicylhydrazide and diarylethene with good photochromic properties.

## Graphical abstract

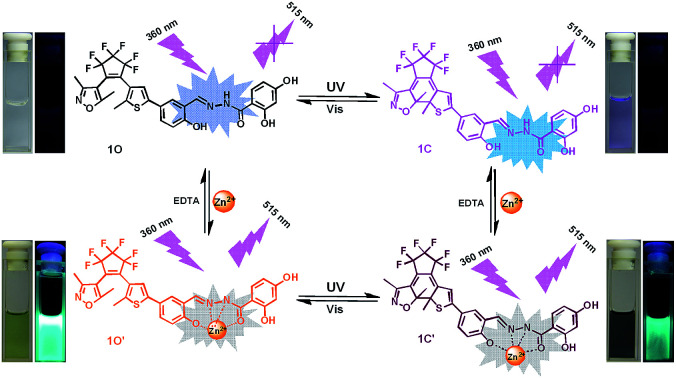

## Introduction

As we all know, zinc(ii) is the second most abundant and essential element after iron ions (Fe^2+^ and Fe^3+^) in the human body and performs a variety of functions.^1–3^ It is an integral part of numerous enzymes and plays a critical role in various biological processes, such as protein metabolism, the immune system, gene transcription and regulation.^4–7^ The research on Zn^2+^ has drawn considerable attention among biologists, chemists, environmentalists and pharmacologists for its chemical and physical properties. The concentration of Zn^2+^ in the human body ranges from nanomolar (nM) to millimolar (mM)^8^ and it is indispensable to living organisms. Depletion of biological Zn^2+^ leads to a decrease in wound health strength as a result of impaired collagen synthesis.^9^ Zn^2+^ is a relatively non-toxic element, while its high level is cytotoxic. Unbalanced metabolism of Zn^2+^ may lead to a series of diseases, such as Alzheimer's disease,^10^ Parkinson's disease,^11^ diabetes,^12^ prostate cancer^13^ and immune dysfunction.^14^ It is important for us to maintain the balance of Zn^2+^ in human body. Therefore, monitoring the distribution and concentration of Zn^2+^ in environmental or biologic samples becomes important.

The demand for chemosensors that are selective and sensitive for specific target ion is continuously increasing. Numerous sensitive detection methods for metal ion recognition have been widely used in the field of analytical chemistry, biology and environmental processes,^15–18^ such as mass spectrometry, atomic absorption spectroscopy and high performance liquid chromatography. However, these methods are laborious and require the use of complex equipment. Fluorescence is a powerful tool to detect target ions for its simplicity, easy implementation, high sensitivity and low detection limit.^19–21^ When a specific fluorescent chemosensor is added to a solution of target metal ion, a color change can be observed accompanied by the changes of fluorescent characteristics. Based on this apparent phenomenon, we can design efficient chemosensors for the recognition of specific ions.

Over the years, many fluorescent chemosensors have been reported for the detection of Zn^2+^. Several Zn^2+^ sensors have been developed based on different fluorophores, such as quinoline,^22–24^ fluorescein,^25–28^ coumarin,^29–32^ peptide,^33^ and pyrene.^34^ However, most of them lack the smartness in Zn^2+^ selectivity, sensitivity and interference resistance from Cd^2+^ ion,^35,36^ or there is a low yield resulting from complex purification protocols.^37–39^ As shown in Table 1, most of these sensors can not distinguish Zn^2+^ from Cd^2+^.^40–44^ Zn^2+^ and Cd^2+^ are located in the same group of the periodic table and show similar photo-physical changes in these sensors.^45^ So, an active search of new analytic agents with higher sensitivity and selectivity for Zn^2+^ continues at the present time.

**Table 1:** Comparative study of the analytical performance of 1O with other reported sensors

Ref.	Structure	LOD (μM)	Stokes shift (nm)	Solvent concent	Interferents
This Work	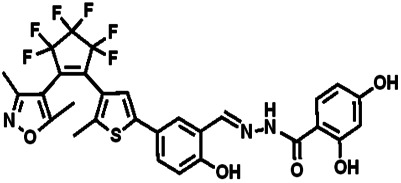	1.28	65	THF	None
39	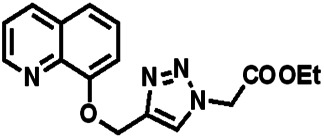	99.1	128	CH_3_CN(5%)	Cd^2+^
40	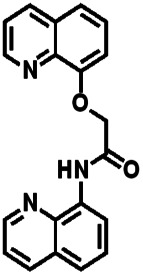	—	128	CH_3_CN	Cd^2+^
41	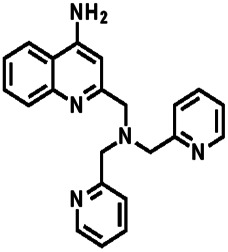	0.198	42	CH_3_CN(10%)	Cd^2+^
42	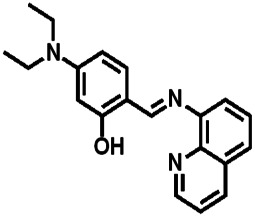	1.85	110	CH_3_CN(90%)	Mg^2+^
43	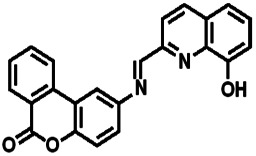	10	75	DMF	Cd^2+^

Photo-stimuli responsive materials have attracted much attention due to their potential applications in optical devices, controlled release, clean energy, sensors, *etc.* Until now, plenty of materials based on different photoactive groups have been explored. Spiropyran, spirooxazine, diarylethene and fulgide derivatives are photochromic materials based on photo-induced isomerization involving ring-opening/closing reactions.^46,47^ Photochromism refers to a reversible change in the properties of a molecule in response to light. Among the various photochromic compounds, diarylethenes are gaining increasing attention in the field of photo-electronics, such as optical memory media and photo-switching devices, due to their high thermal stability, excellent fatigue resistance, and characteristic bistability.^48,49^

**Scheme 1:**
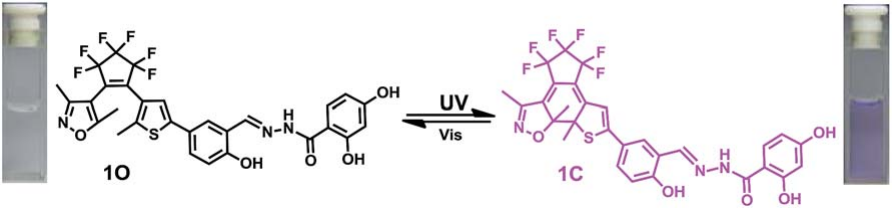
Synthesis route to diarylethene 1O.

In the current work, we reported a Zn^2+^ chemosensor with a 2,4-dihydroxybenzoyl hydrazine unit. Salicylhydrazide is one of the important members in Schiff base family because it offers a number of possibilities for different modes of coordination with transition metal ions.^50,51^ On the other side, considering the advantages of fast response and excellent thermal stability for diarylethene derivatives, we designed and synthesized the compound 1O. The chemosensor detected Zn^2+^ with high selectivity and specificity accompanied by obvious color changes by stimuli of lights and metal ions. Besides, addition of Zn^2+^ into the compound 1O resulted in a change in the absorbance spectra, making 1O a naked-eye detector for Zn^2+^. The photochromic process of the diarylethene derivate was shown in Scheme 1.

## Experimental

### General methods

All solvents were of analytical purity and were purified by distillation before use. Other reagents were used without further purification. Mass spectra were measured with a Bruker amazon SL Ion Trap Mass spectrometer. ^1^H NMR and ^13^C NMR spectra were recorded on a Bruker AV400 (400 MHz) spectrometer with tetramethylsilane as an internal standard. Infrared spectra (IR) were recorded on a Bruker Vertex-70 spectrometer. Melting point was measured on a WRS-1B melting point apparatus. Fluorescence spectra were measured using a Hitachi F-4600 spectrophotometer. The fluorescence quantum yield was measured with an Absolute PL Quantum Yield Spectrometer QY C11347-11. Absorption spectra were measured using an Agilent 8453 UV/vis spectrophotometer with an MUL-165 UV lamp and a MVL-210 visible lamp as equipments of photoirradiation. The solutions of metal ions (0.1 mol L^−1^) were prepared by the dissolution of their respective metal nitrates in distilled water, except for K^+^, Ba^2+^, Mn^2+^, and Hg^2+^ (all of their counter ions were chloride ions). Necessary dilutions were made according to each experimental set up. All of the measurements were conducted at room temperature unless otherwise stated.

### Synthesis of 1O

**Scheme 2:**
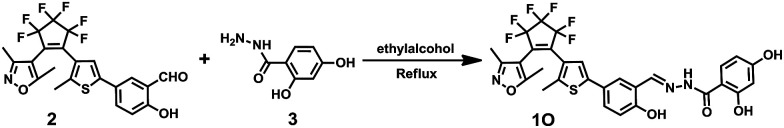
Synthesis route to diarylethene 1O.

Diarylethene 1O was synthesized as presented in Scheme 2. Compound 2 was synthesized according to the previous reported similar method.^58^ Compound 2 (0.25 g, 0.5 mmol) and 2,4-dihydroxybenzoyl hydrazine (0.1 g, 0.6 mmol) were dissolved in a round-bottom flask with ethanol (20 mL). After refluxed for 4 h until no compound 2 was detected by the TLC silica gel plate. The mixture was cooled to room temperature and concentrated under vacuum. The crude product was purified by recrystallization with ethanol to give compound 1O (0.29 g, 0.46 mmol) as a white solid in 83% yield. Mp 458–459 K; ^1^H NMR (400 MHz, DMSO-*d*_*6*_, TMS), *δ* (ppm): 2.00 (s, 3H), 2.06 (s, 3H), 2.28 (s, 3H), 6.35 (s, 1H), 6.40 (d, 1H, *J* = 8.0 Hz), 7.01 (d, 1H, *J* = 8.0 Hz), 7.39 (s, 1H), 7.57–7.60 (m, 1H), 7.82 (d, 2H, *J* = 8.0 Hz), 8.69 (s, 1H), 10.33 (s, 1H), 11.45 (s, 1H), 11.96 (s, 1H), 12.19 (s, 1H). (Fig. S1, ESI†) ^13^C NMR (DMSO-*d*_*6*_, 100 MHz): 10.13, 11.69, 13.98, 102.70, 102.87, 103.93, 107.06, 107.62, 117.29, 119.40, 121.33, 124.05, 124.12, 125.75, 128.57, 129.87, 140.42, 141.81, 147.25, 157.44, 157.06, 162.04, 162.87, 170.16. (Fig. S2, ESI†) IR (KBr, *ν*, cm^−1^): 601, 982, 1064, 1136, 1279, 1612, 3255. (Fig. S3, ESI†). HR-MS (ESI, *m*/*z*): [M − H]^−^ calcd for (C_29_H_20_F_6_N_3_O_5_S)^−^, 636.1028; found 636.1034 (Fig. S4, ESI†).

## Results and discussion

### Photochromic and fluorescent behaviors

**Fig. 1:**
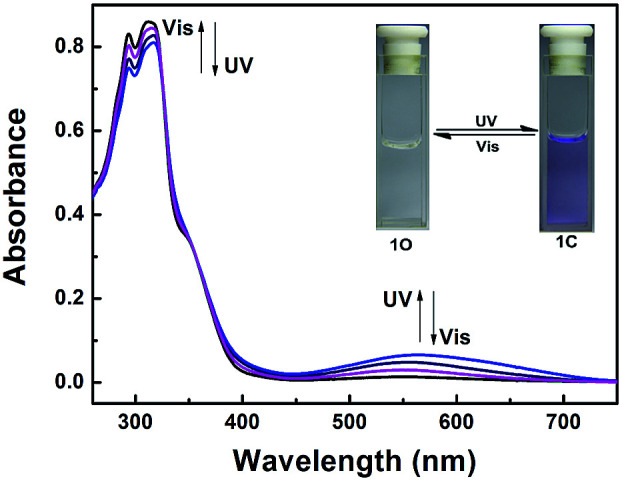
Absorption spectra and color changes of 1O by photoirradiation.

The absorption spectrum and fluorescence changes of 1O were measured in tetrahydrofuran (2.0 × 10^−5^ mol L^−1^) at room temperature. As shown in Fig. 1, the absorption maximum of 1O was observed at 312 nm (*ε* = 4.30 × 10^4^ mol^−1^ L cm^−1^), which was resulted from π–π* transition.^52^ On irradiation with 297 nm light, a new absorption band centered at 565 nm (*ε* = 3.29 × 10^3^ mol^−1^ L cm^−1^) appeared and the color of 1O solution turned purple due to the formation of closed-ring isomer 1C. At the same time, the absorption band peaked at 312 nm decreased gradually. Reversely, the purple solution could be completely bleached upon irradiation with visible light (*λ* > 500 nm) and its absorption spectrum recovered to that of the open-ring isomer 1O. Fig. S5† showed the emission spectral changes of 1O by alternating irradiation with UV and visible light. When excited with 365 nm light, an emission peak of 1O was observed at 580 nm. The fluorescence quantum yield of 1O was determined to be 0.006. On irradiation with 297 nm light, the photocyclization happened and its emission intensity decreased slightly due to the formation of non-fluorescent closed-ring isomer 1C. The fluorescence quantum yield of 1C was determined to be 0.004. The emission intensity of 1O was quenched to *ca.* 80% at the photostationary state. This phenomenon indicated that the diarylethene unit exhibited relatively low fluorescent modulation efficiency in tetrahydrofuran and it would provide a low background when act as a Zn^2+^ sensor. The residual fluorescence in the photostationary state might be attributed to the incomplete cyclization reaction and existence of parallel conformations.^53,54^ Back irradiation with appropriate wavelength visible light regenerated its opening isomer and recovered the original emission intensity.

### Absorption changes induced by Zn^2+^/EDTA and UV/vis light

**Fig. 2:**
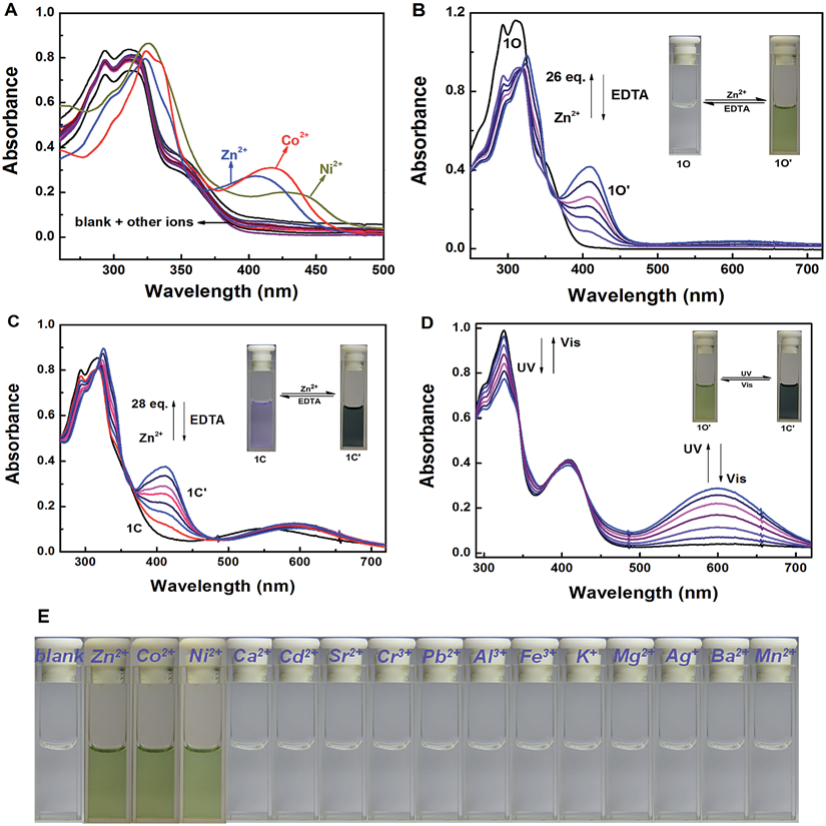
Changes in absorption spectra and color of 1O induced by Zn^2+^/EDTA and light in tetrahydrofuran (2.0 × 10^−5^ mol L^−1^): (A) changes in absorption spectra on addition of different metal ions (B) 1O induced by Zn^2+^/EDTA, (C) 1C induced by Zn^2+^/EDTA, (D) 1O′ induced by UV/vis light, (E) color changes on addition of different metal ions.

The absorption property of 1O was investigated in tetrahydrofuran (2.0 × 10^−5^ mol L^−1^) at room temperature. As shown in Fig. 2A, when different metal ions (5 equiv. 0.1 mol L^−1^) were added to the solution of 1O, including Zn^2+^, Al^3+^, Fe^3+^, Cr^3+^, K^+^, Ba^2+^, Ca^2+^, Ni^2+^, Mg^2+^, Mn^2+^, Cd^2+^, Sr^2+^, Co^2+^, Pb^2+^ and Ag^+^, the absorption spectral had no obvious changes except Zn^2+^, Ni^2+^ and Co^2+^. New absorption bands centered at 408 nm (*ε* = 2.09 × 10^4^ mol^−1^ L cm^−1^), 438 nm (*ε* = 1.25 × 10^4^ mol^−1^ L cm^−1^) and 420 nm (*ε* = 1.60 × 10^4^ mol^−1^ L cm^−1^) were observed respectively. The colorless 1O solution turned olive on addition of Zn^2+^, Ni^2+^ and Co^2+^ over other metal ions (Fig. 2E). The above results indicated that 1O could be used as a detective colorimetric sensor for Zn^2+^. But the selectivity is not so good with the interference of Ni^2+^ and Co^2+^.

Under the same experiment conditions, we studied the optimal algorithm of 1O induced by Zn^2+^ and UV/vis light. As shown in Fig. 2B, with the addition of 26 equiv. of Zn^2+^ (0.1 mol L^−1^) to the solution of 1O, a new absorption band centered at 408 nm appeared with the concomitant color change from colorless to olive due to the formation of 1O–Zn^2+^ complex (1O′) with a much steadier rigid construction than 1O. When Zn^2+^ was added to the solution of 1C, the absorbance at 410 nm (*ε* = 1.88 × 10^4^ mol^−1^ L cm^−1^) increased, at the same time, the 565 nm absorbance was red-shifted by 35 nm (Fig. 2C). The phenomena might be on account of formation of the 1C–Zn^2+^ complex (1C′). Then, by addition of excess EDTA (0.1 mol L^−1^) to the solution of 1O′ or 1C′, the absorption band all recovered to 1O or 1C because EDTA possibly stripped Zn^2+^ away from the cavity by the binding zone. Furthermore, as shown in Fig. 2D, when the maximum absorption band of 1O′ was reached, upon irradiation with 297 nm UV light, a new absorption band appeared clearly which centered at 600 nm (*ε* = 1.44 × 10^4^ mol^−1^ L cm^−1^) and the color changed from olive to dark slate gray for the formation of the closed-ring isomer 1C′. Meanwhile, it could return back to the open-ring isomer 1O′ on irradiation with visible light (*λ* > 500 nm), indicating that the open-ring isomer 1O′ and the closed-ring isomer 1C′ reaction was reversible.

### Fluorescence response to metal ions

**Fig. 3:**
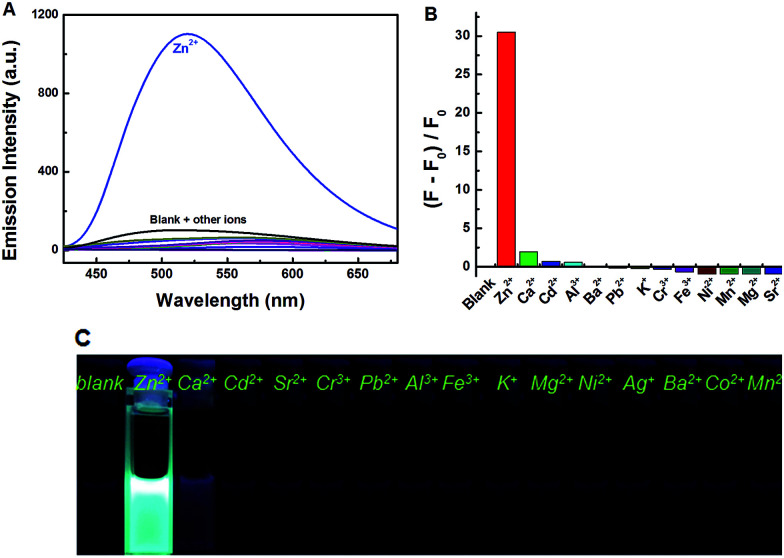
Fluorescence changes of 1O induced by the addition of various metal ions (5.0 equiv.): (A) emission intensity in tetrahydrofuran; (B) emission intensity; (C) photos of fluorescence.

Under the same experimental conditions, Fig. 3 showed the emission spectral and fluorescence color changes of 1O induced by various metal ions (5 equiv. 0.1 mol L^−1^) such as Zn^2+^, Al^3+^, Fe^3+^, Cr^3+^, K^+^, Ba^2+^, Ca^2+^, Ni^2+^, Mg^2+^, Mn^2+^, Cd^2+^, Sr^2+^, Co^2+^, Pb^2+^ and Ag^+^. We found that 1O can detect Zn^2+^ in tetrahydrofuran (2.0 × 10^−5^ mol L^−1^). The fluorescence of 1O was notably changed when Zn^2+^ was added, while the addition of other cations caused no obvious changes (Fig. 3A). When Zn^2+^ was added to the solution of 1O, the fluorescence intensity was enhanced evidently and the emission peak was blue shifted from 580 nm to 515 nm with a concomitant fluorescent color change from dark to bright aliceblue caused by the formation of 1O′. The results showed that there was no interference of Cd^2+^ or Mg^2+^, indicating good selectivity of Zn^2+^. Therefore, 1O can be used as an efficient fluorescence chemosensor for Zn^2+^ recognition.

**Fig. 4:**
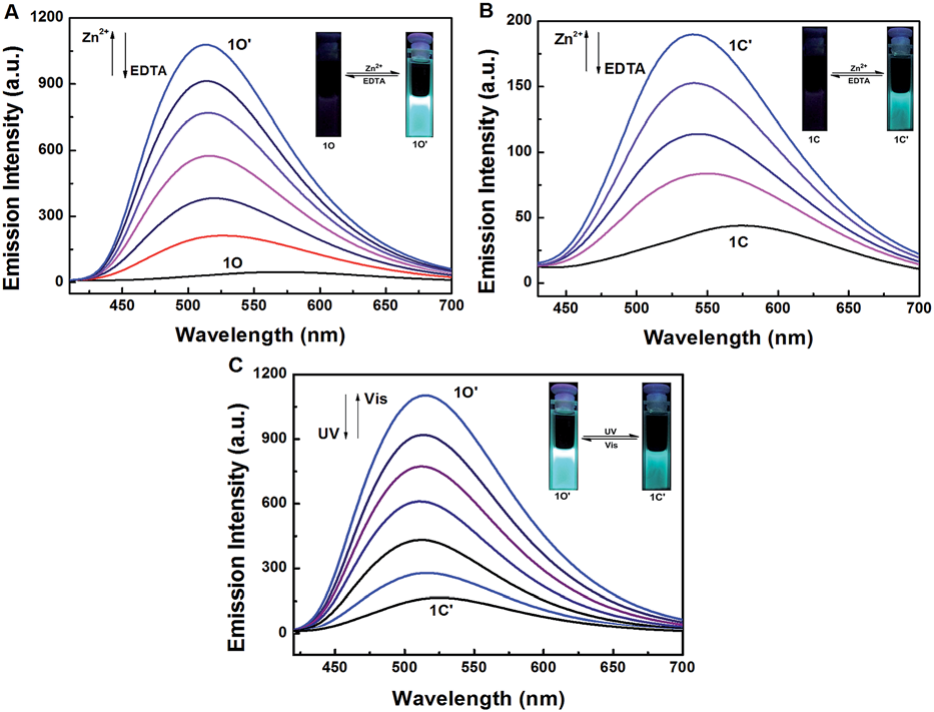
Changes in fluorescence and color of 1O induced by Zn^2+^/EDTA and light in tetrahydrofuran (2.0 × 10^−5^ mol L^−1^): (A) 1O induced by Zn^2+^/EDTA, (B) 1C induced by Zn^2+^/EDTA, (C) 1O′ triggered by light.

To further evaluate the responsive nature of 1O induced by Zn^2+^, a series of fluorescence titration tests were carried out in tetrahydrofuran (2.0 × 10^−5^ mol L^−1^) at room temperature. When Zn^2+^ was added to the solution of 1O from 0 to 18 equiv., the fluorescence intensity increased significantly with a blue shift of 65 nm from 580 nm to 515 nm (Fig. 4A). The titration experiment was shown in Fig. S6.† Compared with 1O, the fluorescence intensity was enhanced by 31 fold. The increased emission intensity and blue shift could be ascribed to the formation of 1O′. The fluorescent quantum yield of 1O′ was determined to be 0.044. The stable chelation of 1O with Zn^2+^ inhibited the C=N isomerization and led to a rigid fluorophore structure, causing enhanced fluorescence intensity.^55,56^ The fluorescence spectrum of 1O′ recovered to that of 1O by adding an aqueous solution of excess EDTA (0.1 mol L^−1^) which possibly strips Zn^2+^ away from the cavity by the binding zone, indicating that the complexation–decomplexation reaction between 1O and Zn^2+^ was reversible. Similar to 1O, the fluorescence of 1C could also be effectively modulated by Zn^2+^ in tetrahydrofuran. After adding 8.0 equiv. of Zn^2+^ to the solution of 1C, its fluorescence intensity was enhanced by 4 folds and the emission peak was blue-shifted from 580 to 530 nm due to the formation of 1C′ (Fig. 4B). We also investigated the photochromism of 1O′. As shown in Fig. 4C, when the maximum intensity was reached, upon irradiation with 297 nm UV light, the emission intensity of 1O′ was quenched to *ca.* 15% due to the formation of 1C′, the fluorescent quantum yield of 1C′ was determined to be 0.028. It can come back to that of 1O′ by irradiation with appropriate visible light and followed by a color change from bright aliceblue to powderblue. The irradiation with UV/vis light reaction between 1O′ and 1C′ was also reversible.

**Fig. 5:**
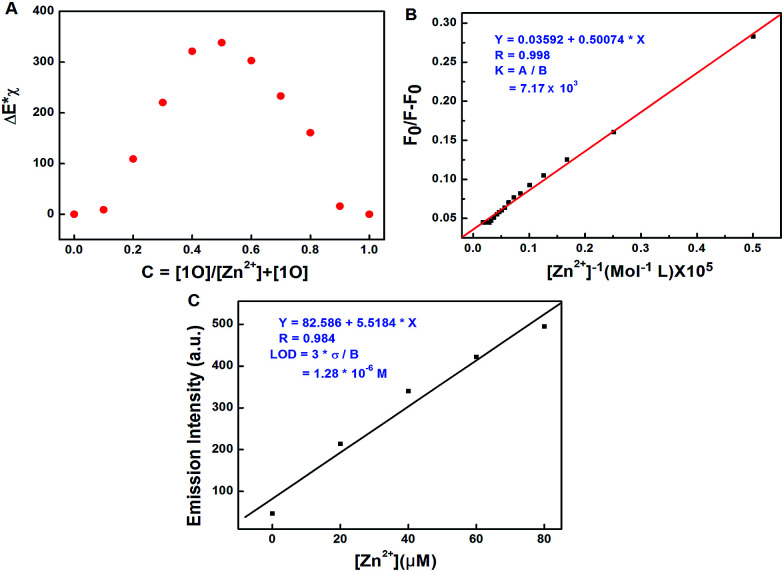
(A) Job's plot showing the 1 : 1 complex of 1O–Zn^2+^ in tetrahydrofuran, (B) Hildebrand–Benesi plot based on the 1 : 1 for 1O, the binding constant of 1O with Zn^2+^ was calculated to be 7.17 × 10^3^ L mol^−1^, (C) the limit of detection (LOD), LOD is 1.28 × 10^−6^ mol L^−1^.

**Scheme 3:**
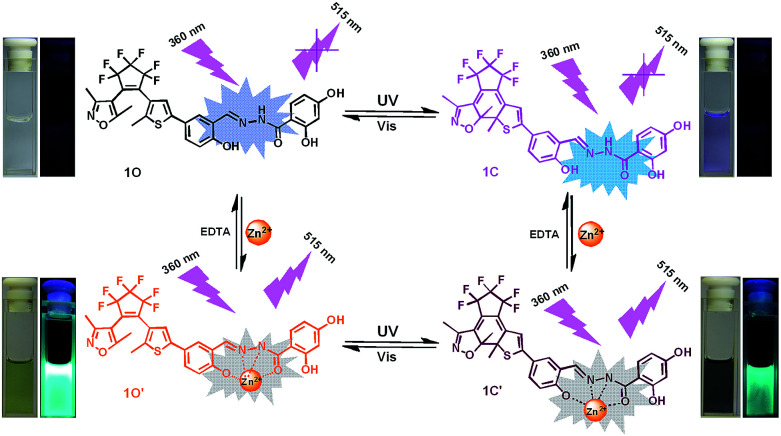
Photochromism, color and fluorescence changes of 1O induced by Zn^2+^/EDTA and lights.

To investigate the coordination mode of 1O with Zn^2+^, Job's plot analysis was performed according to the reported method.^57^ As shown in Fig. 5A, the maximum value was achieved when the molar fraction of [1O]/([1O] + [Zn^2+^]) was about 0.5, demonstrating a 1 : 1 stoichiometry between 1O and Zn^2+^. Based on the 1 : 1 stoichiometry and fluorescence titration data, the association constant (*K*_a_) of 1O with Zn^2+^ was calculated from the slope and intercept of the linear plot to be 7.17 × 10^3^ L mol^−1^ (*R* = 0.984) (Fig. 5B). The detection limit was calculated to be 1.28 × 10^−6^ mol L^−1^ for Zn^2+^ (Fig. 5C). To further confirm the coordination mode of 1O and Zn^2+^, ESI mass spectra were recorded. The ESI-MS peak at 699.1031 assigned to [1O + Zn^2+^ − 3H]^−^ (calcd 699.0241) was observed (Fig. S7†), providing strong evidence for the formation of a 1 : 1 complex between 1O and Zn^2+^. The proposed binding mode between 1O and Zn^2+^ was shown in Scheme 3.

### Application in logic circuit

**Fig. 6:**
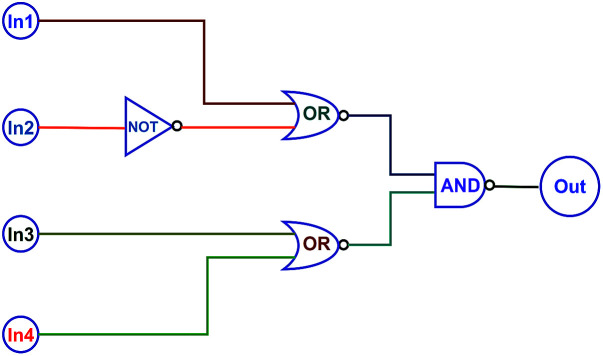
The combinational logic circuits equivalent to the truth table give in Table 1, In1 (297 nm UV light), In2 (*λ* > 500 nm light), In3 (Zn^2+^), In4 (EDTA) and output (*λ*_em_ = 515 nm).

On the basis of the fact that the absorption and fluorescent intensity of the target diarylethene could be effectively modulated by Zn^2+^/EDTA and light, a type of logic circuits was constructed by using light irradiation and Zn^2+^/EDTA as the input signals. The fluorescence at 515 nm was used as an output signal. As shown in Fig. 6, the photochromic behaviors of 1O could be effectively modulated by Zn^2+^/EDTA and UV/vis light. Thus, one logic circuit was constructed by using the combination of four input signals (In1: 297 nm UV light, In2: *λ* > 500 nm visible light, In3: Zn^2+^, and In4: EDTA) and an output (fluorescence emission at 515 nm). The four inputs and one output could be either “on” or “off” state with different Boolean values. When 297 nm light was employed, In1 was switched to “on” state with a Boolean value of “1”. Similarly, In2 was “1” corresponding to irradiation with appropriate visible light (*λ* > 500 nm), In3 was “1” corresponding to the addition of Zn^2+^, and In4 was “1” corresponding to the addition of EDTA. The emission intensity of 1O at 515 nm was regarded as the initial value and when the change of fluorescence intensity at 515 nm was 31 fold larger than the initial value, it was regarded as “on” state with a Boolean value of “1”. Otherwise, it was regarded as “off” state with a Boolean value of “0”. Upon the stimuli of different inputs, the diarylethene exhibited an on–off–on photochromic switching behavior. As a result, 1O could read a string of four inputs and write one output (Table 2).

**Table 2:** Truth table for all possible strings of four binary-input date and the corresponding output digit

Inputs				Output *λ*_em_ = 515 nm
In1 (UV)	In2 (vis)	In3 (Zn^2+^)	In4 (EDTA)	
0	0	0	0	0
1	0	0	0	0
0	1	0	0	0
0	0	1	0	1
0	0	0	1	0
1	1	0	0	0
1	0	1	0	1
1	0	0	1	0
0	1	1	0	1
0	1	0	1	0
0	0	1	1	0
1	1	1	0	0
1	1	0	1	0
1	0	1	1	0
0	1	1	1	0
1	1	1	1	0

## Conclusions

In summary, a highly sensitive fluorescent “turn-on” sensor based on a photochromic diarylethene derivative with a salicylhydrazide unit was developed. It exhibited excellent photochromic properties, high selectivity and specificity toward Zn^2+^ over other metal ions. It could also be used as a naked-eye detector for Zn^2+^. Furthermore, the diarylethene showed excellent fluorescent switching behaviors with distinctive color changes in response to the combinational inputs of light and Zn^2+^. Based on these characteristics, a logic circuit was designed by the fluorescence intensity as the output signal with the inputs of UV/vis lights and Zn^2+^/EDTA. All these results will be helpful for the design and construction of new diarylethene derivatives with multi-addressable states and potential applications in fluorescent sensors for special ions.

## Conflicts of interest

There are no conflicts of interest to declare.

## Supplementary Material

RA-008-C7RA13592K-s001
