# Supplementary material for: An efficient and sensitive chemosensor based on salicylhydrazide for naked-eye and fluorescent detection of Zn2+
Source: RSC Adv. 2018 Feb 12;8(12):6727–32. doi: 10.1039/c7ra13592k (PMC9078340; doi:10.1039/c7ra13592k)
Supplement: RA-008-C7RA13592K-s001 [file RA-008-C7RA13592K-s001.pdf]

Electronic Supplementary Material (ESI) for RSC Advances.
This journal is © The Royal Society of Chemistry 2018

Supporting Information

An efficient and sensitive chemosensor based on salicylhydrazide for naked-eye and fluorescent detection of Zn^{2+}

Zhanglin Shi[#], Yayi Tu[#], Shouzhi Pu*

Jiangxi Key Laboratory of Organic Chemistry, Jiangxi Science and Technology Normal University, Nanchang 330013, China

#These authors contributed equally to this work.

*Corresponding author: pushouzhi@tsinghua.org.cn (S. Pu), Tel. & Fax: +86-791-83831996.

Contents

Fig. S1. ^{1}H NMR spectra of **1O** in DMSO-d_6.

Fig. S2. ^{13}C NMR spectra of **1O** in DMSO-d_6.

Fig. S3. IR spectra of **1O**.

Fig. S4. Mass spectrum of **1O**.

Fig. S5. Changes in fluorescence intensity of **1O** upon irradiation with UV/Vis light.

Fig. S6. The emission intensity curve at 510 nm with the addition of different equiv. Zn^{2+}.

Fig. S7. Mass spectrum of **1O′**.

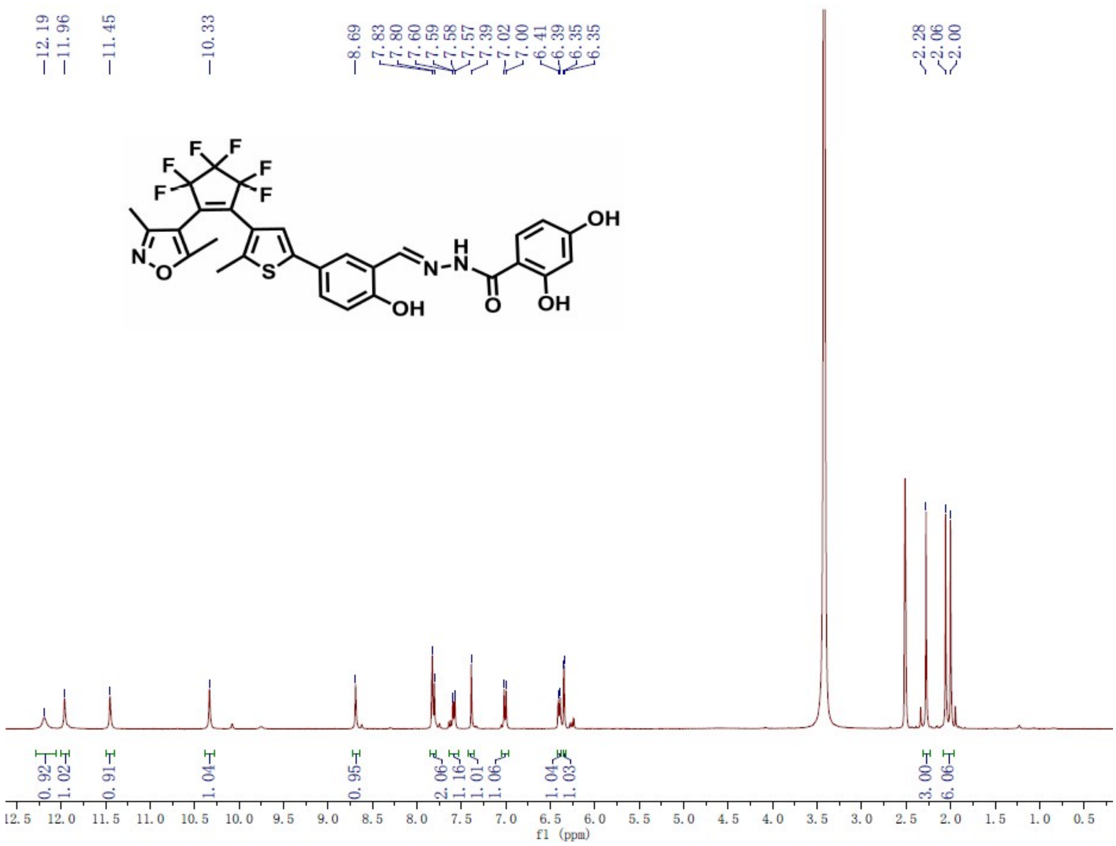

Fig. S1. ^{1}H NMR spectra of **1O** in DMSO-d_6.

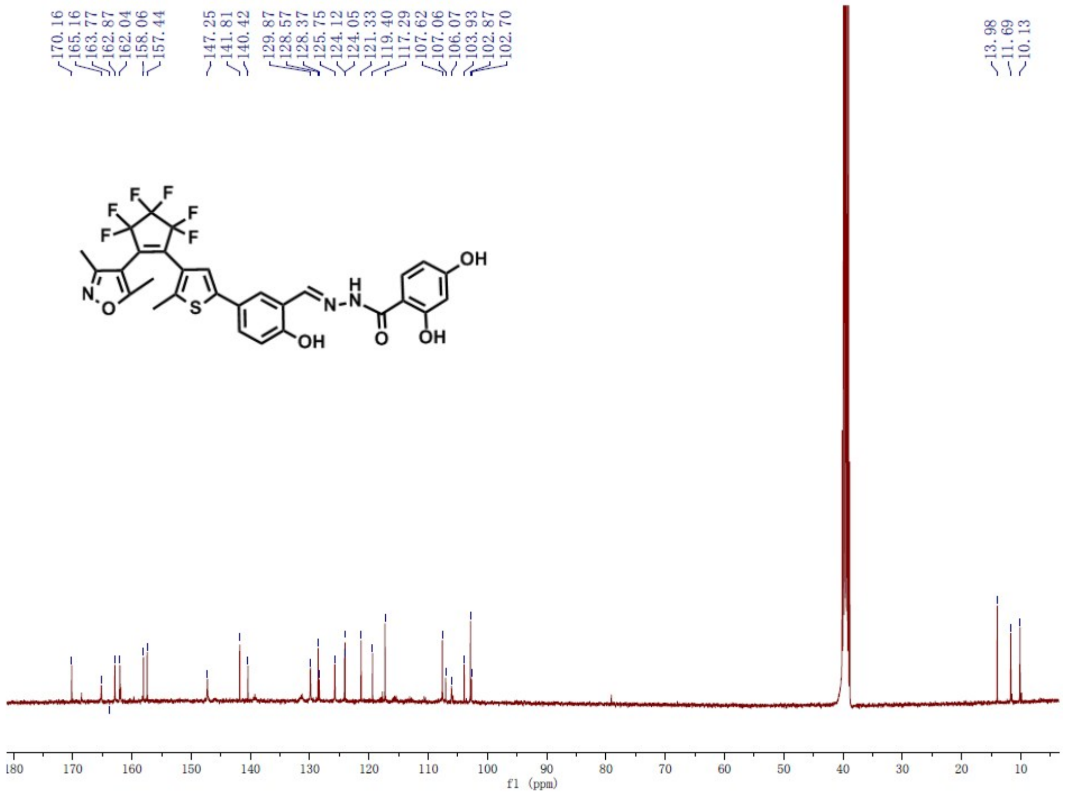

Fig. S2. ^{13}C NMR spectra of **1O** in DMSO-d_6.

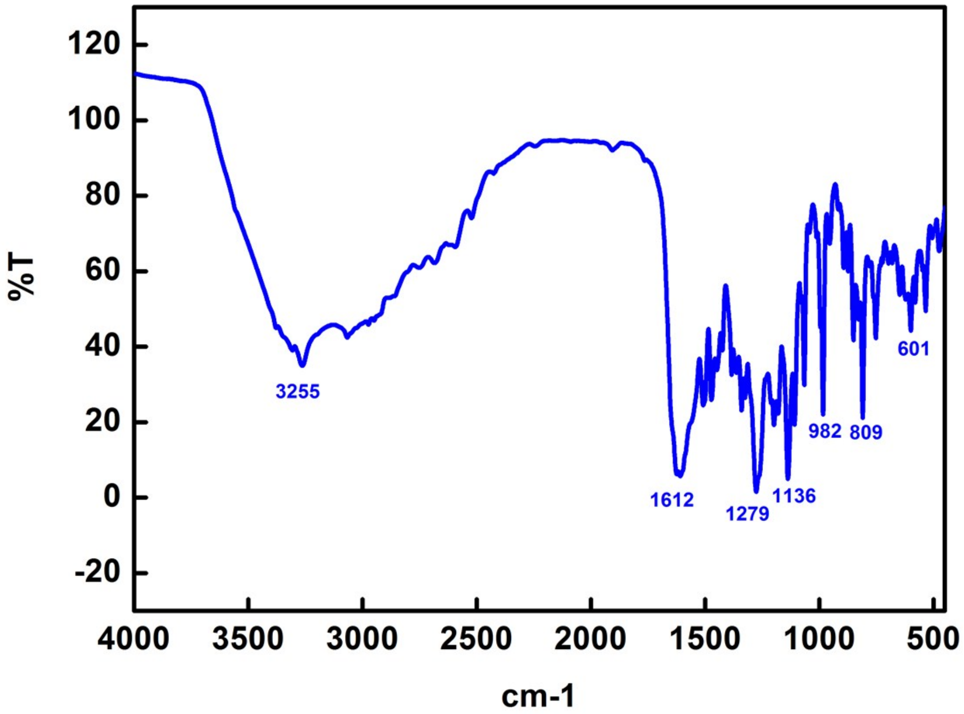

Fig. S3. IR spectra of **1O**.

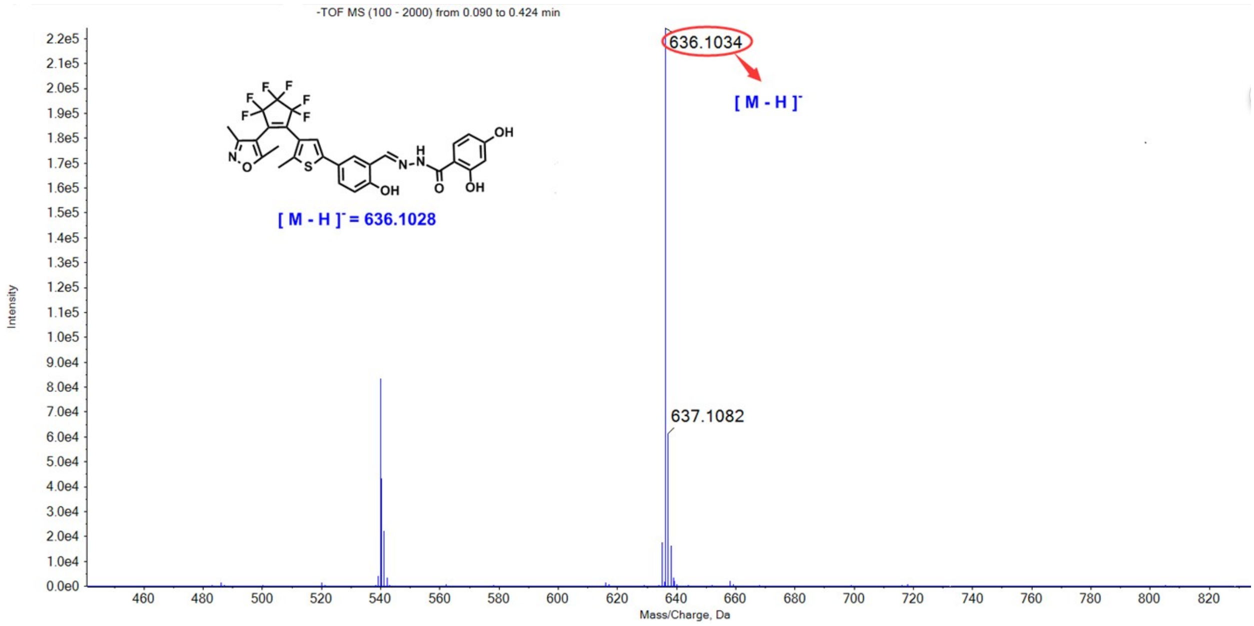

Fig. S4. Mass spectrum of **1O**.

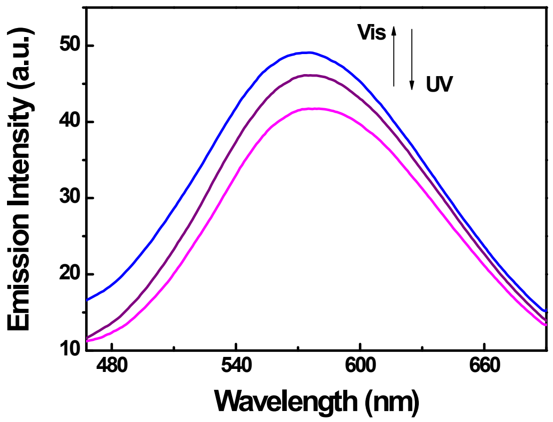

Fig. S5. Changes in fluorescence intensity of **1O** upon irradiation with UV/Vis light.

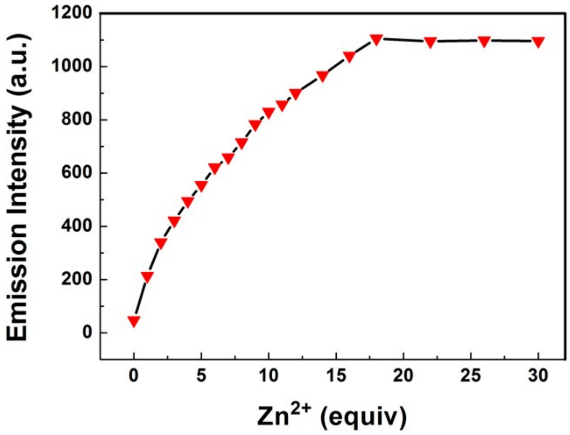

Fig. S6. The emission intensity curve at 510 nm with the addition of different equiv. Zn^{2+}.

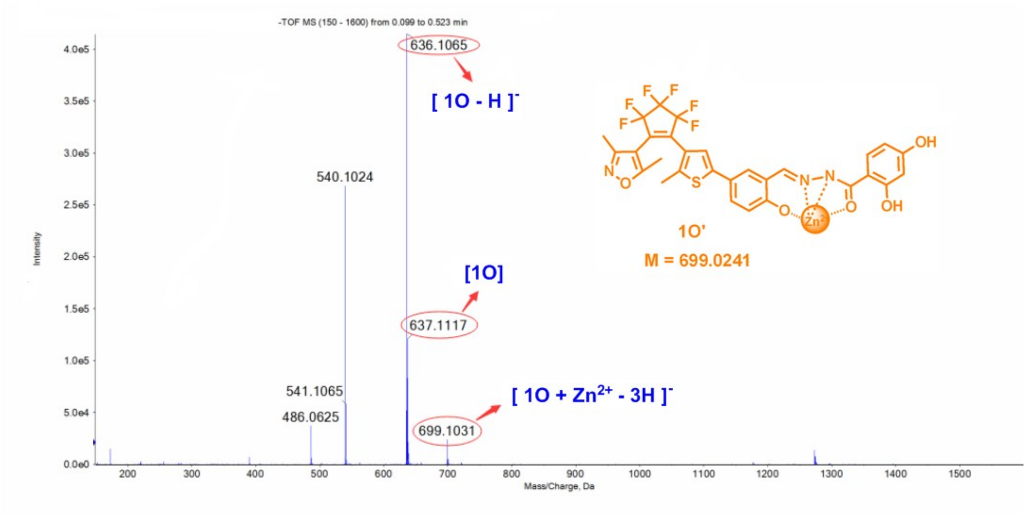

Fig. S7. Mass spectrum of **1O′**.
